# Supplementary material for: GRK specificity and Gβγ dependency determines the potential of a GPCR for arrestin-biased agonism
Source: Commun Biol. 2024 Jul 3;7:802. doi: 10.1038/s42003-024-06490-1 (PMC11220067; doi:10.1038/s42003-024-06490-1)
Supplement: Supplementary file 2 — Supplementary Information [file 42003_2024_6490_MOESM2_ESM.pdf]

GRK specificity and Gβγ dependency determines the potential of a GPCR for arrestin-biased agonism

Edda S. F. Matthees*, Jenny C. Filor*, Natasha Jaiswal, Mona Reichel, Noureldine Youssef, Giulia D'Uonnolo, Martyna Szpakowska, Julia Drube, Gabriele M. König, Evi Kostenis, Andy Chevigné, Amod Godbole, Carsten Hoffmann[1]**

* Contributed equally

** Corresponding author: Prof. Dr. Carsten Hoffmann (carsten.hoffmann@med.uni-jena.de)

Supplementary material

Including Suppl. Fig. 1-9 and Suppl. Tab. 1-14, as well as the respective legends

Supplementary Figure 1

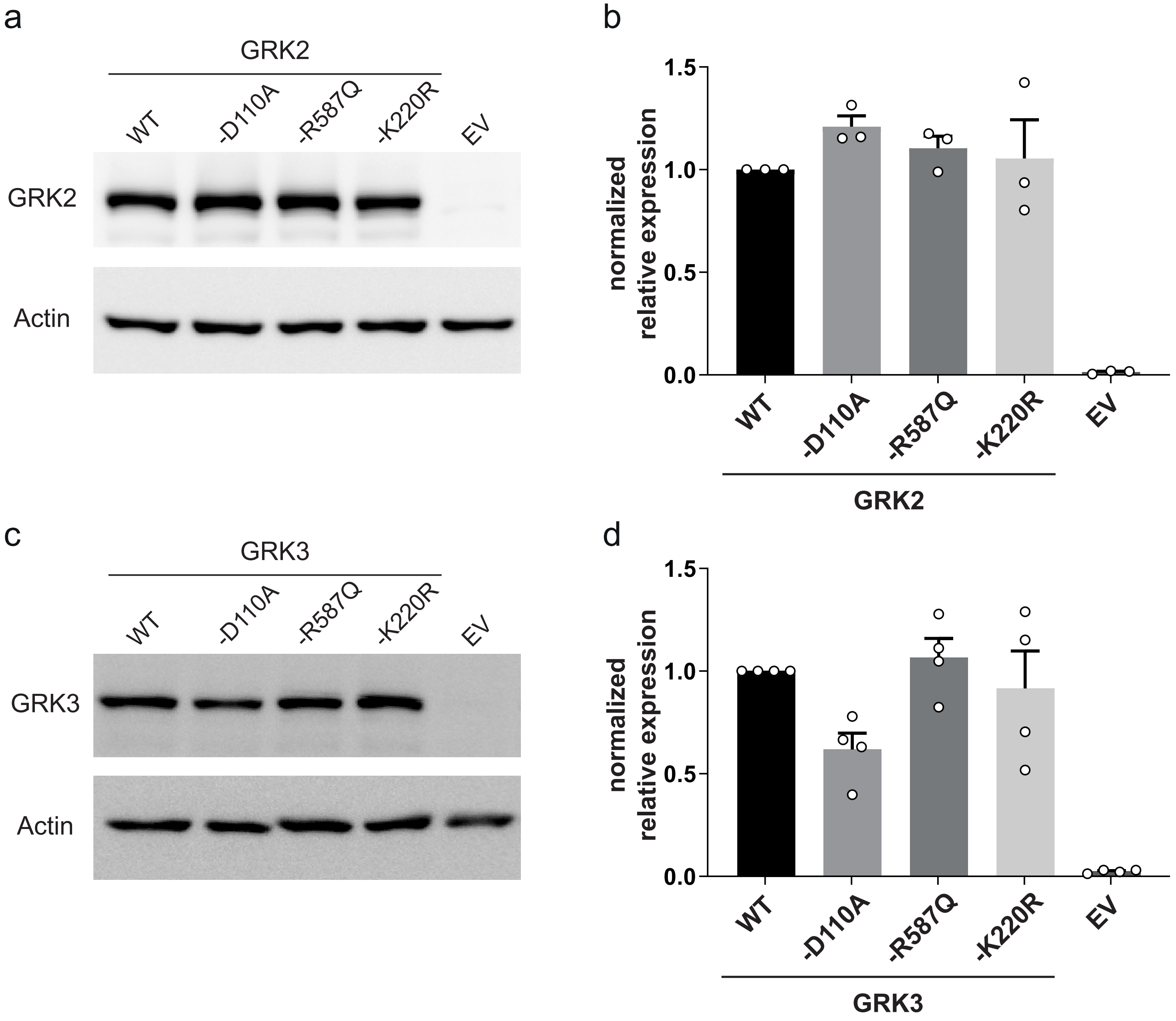

Supplementary Figure 1: Quantification of GRK2/3 and respective mutant constructs expression. a, c, Representative Western blot of *n*=3 (GRK2, a) or *n*=4 (GRK3, c) independent experiments, showing the overexpression of respective GRK2 or GRK3 versions: WT GRK, GRK-D110A, GRK-R587Q and GRK-K220R, in ΔGRK2 (GRK2) or ΔGRK2/3 (GRK3) cells, compared to empty vector (EV) in absence of the ubiquitously expressed GRK with actin as loading control. **b, d,** Quantification of the WT GRK, GRK-D110A, GRK-R587Q and GRK-K220R expression levels of GRK2 or GRK3 respectively from **a, c.** Each signal was normalized to the background and actin signal, detected on the same membrane. Relative expression data are shown of *n*=3 (GRK2) or *n*=4 (GRK3) independent experiments, respectively normalized to WT GRK2 or GRK3 signal.

Supplementary Figure 2

a

Localization of GRK2 constructs

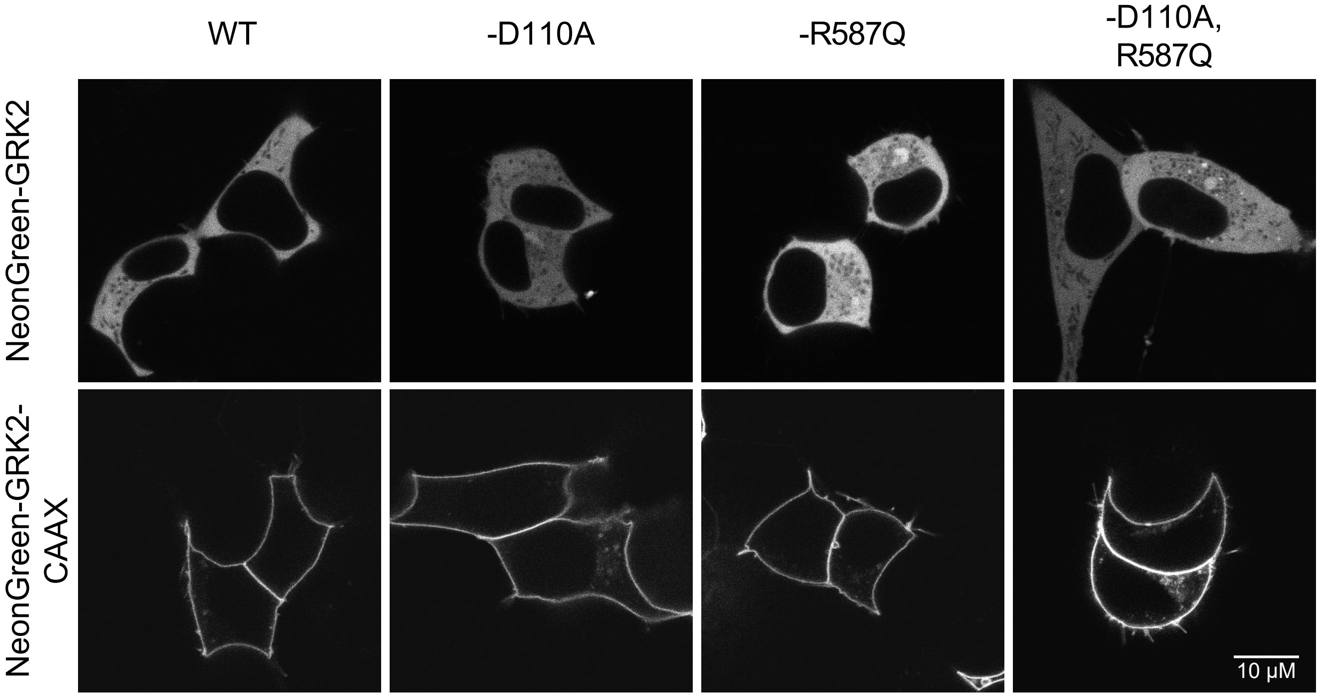

b

Fluorecence intensity of GRK2 constructs

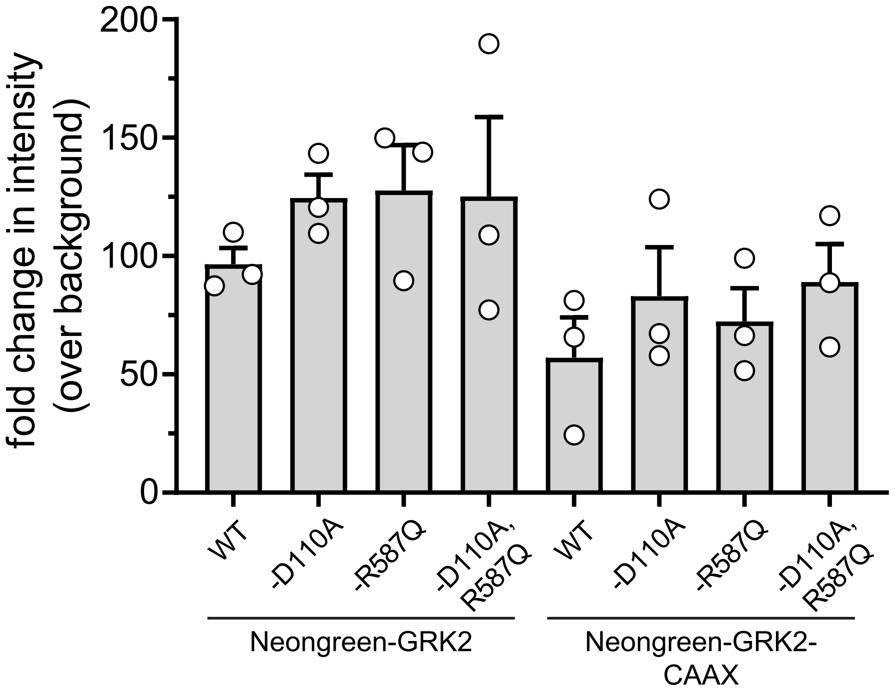

Suppl. Fig. 2: Confirmation of correct localization and expression of utilized GRK2 and mutant constructs. a, ΔQ-GRK cells were transfected with fluorophore-coupled (NeonGreen) GRK2-WT, -D110A, -R587Q, or the double mutant (-D110A, R587Q), as well as the corresponding CAAX-fused versions to ensure the cytosolic or membrane-tethered localization, respectively. Representative confocal images for each utilized GRK2 construct are shown here. **b,** Additionally, fluorescence intensity was measured for all Neongreen-coupled GRK2 constructs. Data are shown as fold change in intensity, normalized to background fluorescence in empty vector (EV)-transfected cells of *n*=3 independent experiments + SEM. Statistical comparison of all conditions are provided in **Suppl. Tab. 1**.

Supplementary Figure 3

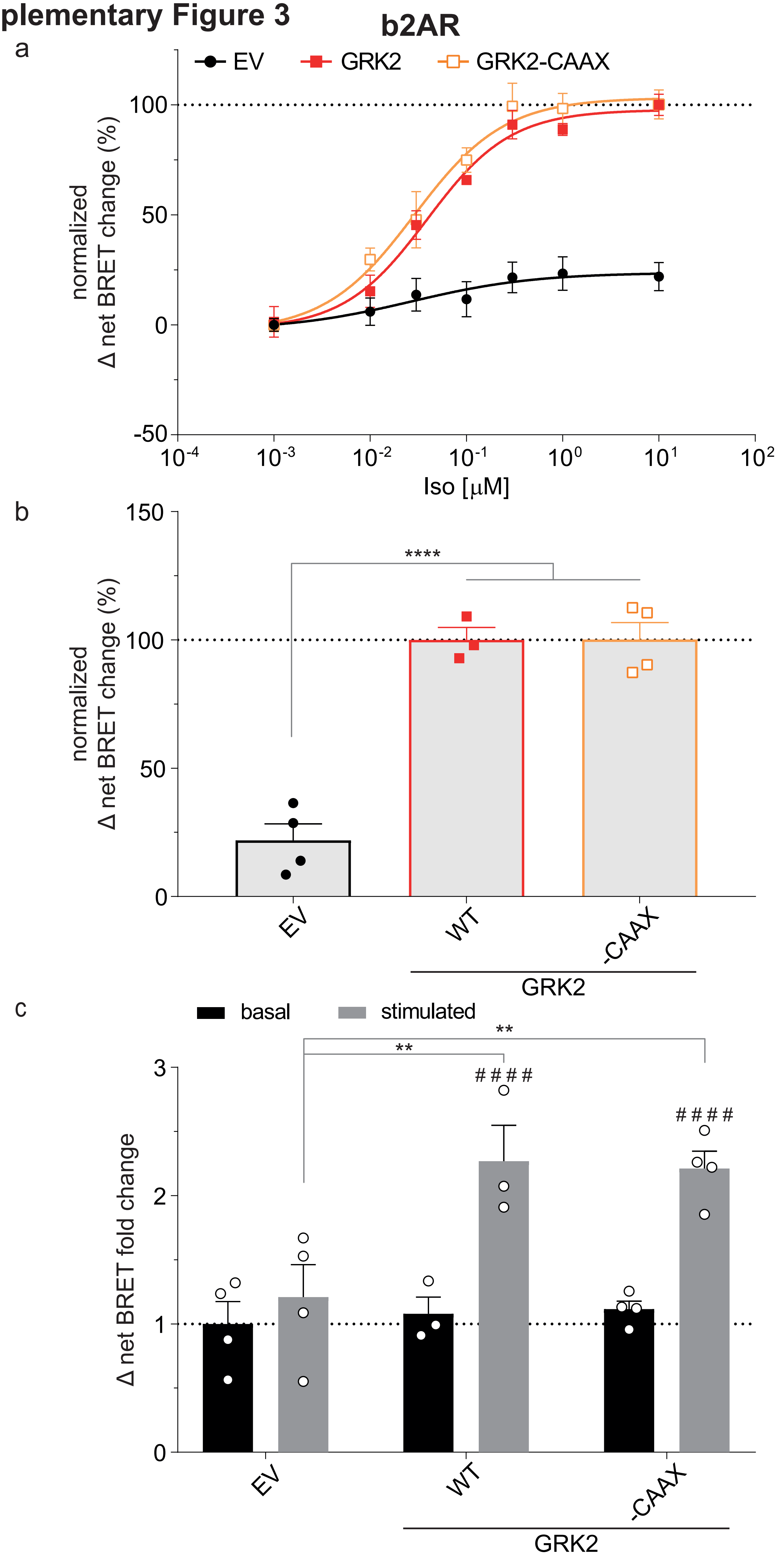

Suppl. Fig. 3: Comparison of GRK2- and GRK2-CAAX-mediated β-arrestin2 recruitment to the b2AR. a, Iso-induced Halo-Tag-β-arrestin2 recruitment to b2AR-NLuc in ΔQ-GRK cells in absence of the ubiquitously expressed GRKs (empty vector (EV)-transfected) and in presence of wild type GRK2 or GRK2-CAAX. Data from **Fig. 1** are shown again to allow a direct comparison of the GRK2 and GRK2-CAAX condition. Data are shown as Δ net BRET change (%) of at least n=3 independent experiments ± SEM, normalized to the maximum response with GRK2. **b,** Normalized BRET data of the highest stimulation of **a** (10 µM Iso) are displayed as bar graphs. Statistical differences were tested using one-way ANOVA, followed by a Tukey's test (**** $p < 0.0001$). **c**, The Halo-corrected mean Δ net BRET fold changes + SEM of the same dataset (not normalized to the highest stimulated value of the GRK2 WT condition) are presented as bar graphs before (basal) and after stimulation with 10 µM Iso (stimulated). The data were normalized to the basal BRET ratio derived from the EV-transfected condition (dotted line). Statistical differences within one condition between basal and stimulated (#) or between the differently transfected conditions (*) were tested using two-way ANOVA, followed by a Sidak's or Tukey's test respectively (** $p < 0.01$; *** $p < 0.001$; ####/**** $p < 0.0001$). All detailed statistical results are provided in **Suppl. Tab. 3**.

Supplementary Figure 4

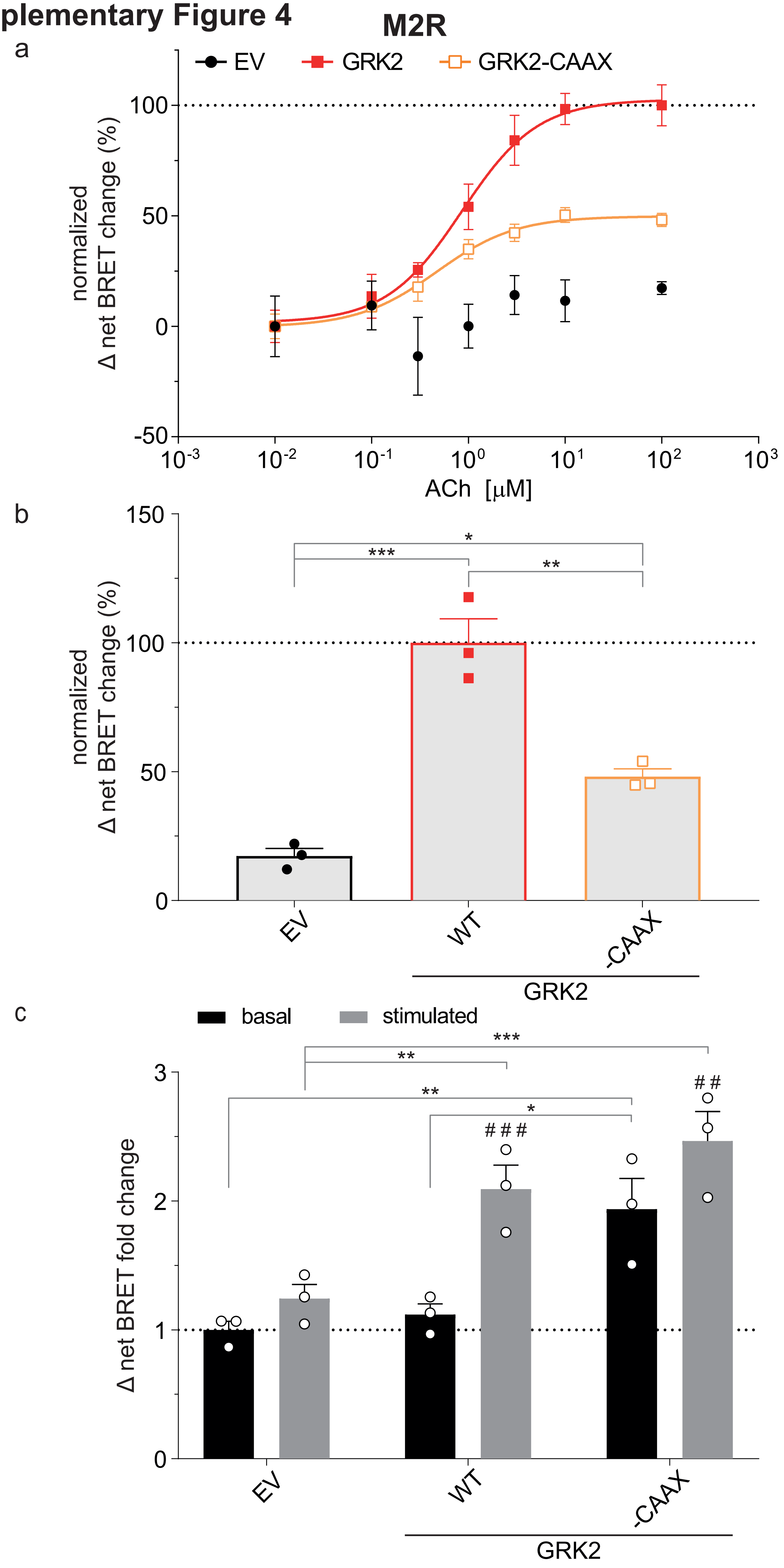

Suppl. Fig. 4: Comparison of GRK2- and GRK2-CAAX-mediated β-arrestin2 recruitment to the M2R. a, ACh-induced Halo-Tag-β-arrestin2 recruitment to M2R-NLuc in ΔQ-GRK cells in absence of the ubiquitously expressed GRKs (empty vector (EV)-transfected) and in presence of wild type GRK2 or GRK2-CAAX. The concentration-response curves of the data in **Fig. 2a, b** are shown to allow a direct comparison of the GRK2 and GRK2-CAAX condition. Data are shown as Δ net BRET change (%) of n=3 independent experiments ± SEM, normalized to the maximum response with GRK2. **b,** Normalized BRET data of the highest stimulation of **a** (100 µM ACh) are displayed as bar graphs and statistical differences were tested using one-way ANOVA, followed by a Tukey's test (* $p < 0.05$; ** $p < 0.01$; *** $p < 0.001$). **c**, The Halo-corrected mean Δ net BRET fold changes + SEM of the same dataset (not normalized to the highest stimulated value of the GRK2 WT condition) are presented as bar graphs before (basal) and after stimulation with 100 µM ACh (stimulated). The data were normalized to the basal BRET ratio derived from the EV-transfected condition (dotted line). Statistical differences within one condition between basal and stimulated (#) or between the differently transfected conditions (*) were tested using two-way ANOVA, followed by a Sidak's or Tukey's test respectively (* $p < 0.05$; ##/** $p < 0.01$; ###/*** $p < 0.001$; **** $p < 0.0001$). All detailed statistical results are provided in **Suppl. Tab. 5**.

Supplementary Figure 5

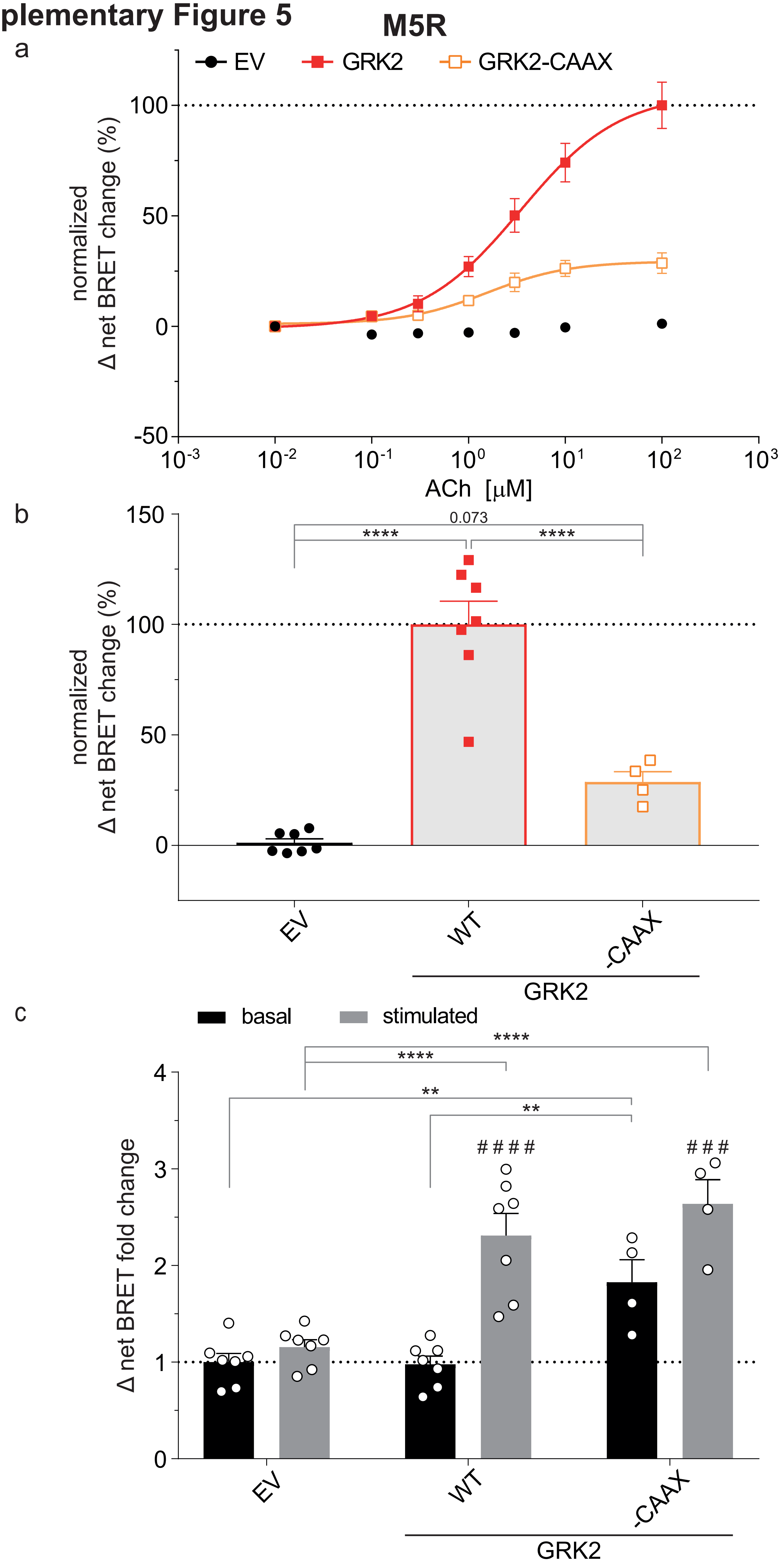

Suppl. Fig. 5: Comparison of GRK2- and GRK2-CAAX-mediated β-arrestin2 recruitment to the M5R. a, ACh-induced Halo-Tag-β-arrestin2 recruitment to M5R-NLuc in ΔQ-GRK cells in absence of the ubiquitously expressed GRKs (empty vector (EV)-transfected) and in presence of wild type GRK2 or GRK2-CAAX. The concentration-response curves of the data in **Fig. 2c, d** are shown to allow a direct comparison of the GRK2 and GRK2-CAAX condition. Data are shown as Δ net BRET change (%) of n=3 independent experiments ± SEM, normalized to the maximum response with GRK2. **b,** Normalized BRET data of the highest stimulation of **a** (100 µM ACh) are displayed as bar graphs and statistical differences were tested using one-way ANOVA, followed by a Tukey's test (**** $p < 0.0001$). **c,** The Halo-corrected mean Δ net BRET fold changes + SEM of the same dataset (not normalized to the highest stimulated value of the GRK2 WT condition) are presented as bar graphs before (basal) and after stimulation with 100 µM ACh (stimulated). The data were normalized to the basal BRET ratio derived from the EV-transfected condition (dotted line). Statistical differences within one condition between basal and stimulated (#) or between the differently transfected conditions (*) were tested using two-way ANOVA, followed by a Sidak's or Tukey's test respectively (** $p < 0.01$; ###/*** $p < 0.001$; ####/**** $p < 0.0001$). All detailed statistical results are provided in **Suppl. Tab. 7**.

Supplementary Figure 6

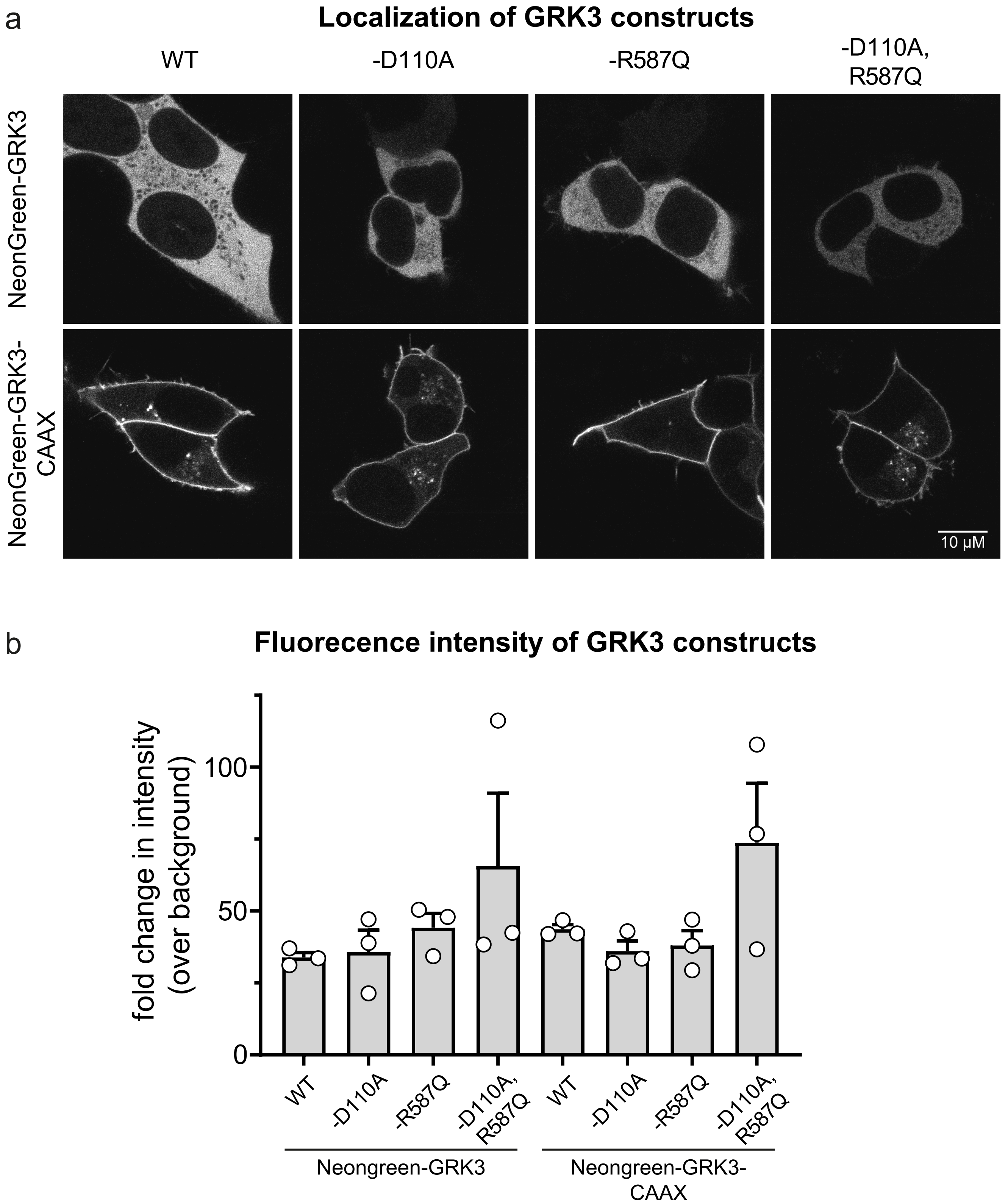

Suppl. Fig. 6: Confirmation of correct localization and expression of utilized GRK3 and mutant constructs. a, Analogously to GRK2, ΔQ-GRK cells were transfected with fluorophore-coupled (NeonGreen) GRK3-WT, -D110A, -R587Q, or the double mutant (-D110A, R587Q), as well as the corresponding CAAX-fused versions to ensure the cytosolic or membrane-tethered localization, respectively. Representative images for each utilized GRK3 construct are shown here. **b,** Fluorescence intensity was measured for all Neongreen-coupled GRK3 constructs. Data are shown as fold change in intensity, normalized to background fluorescence in empty vector-transfected cells, of *n*=3 independent experiments + SEM. Statistical comparison of all conditions are provided in **Suppl. Tab. 8**.

Supplementary Figure 7

b2AR

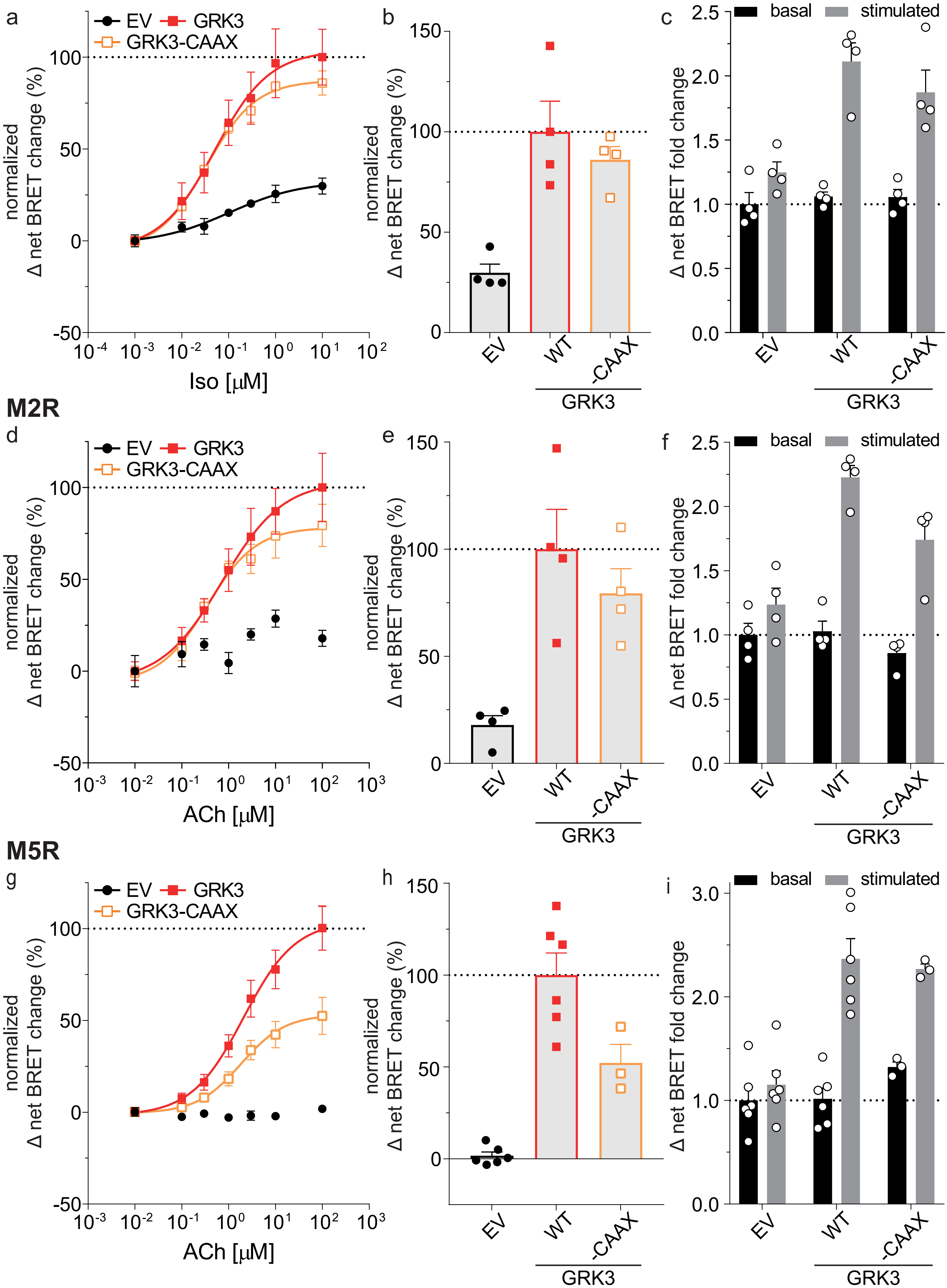

Suppl. Fig. 7: Comparison of GRK3- and GRK3-CAAX-mediated β-arrestin2 recruitment to the b2AR, M2R and M5R. a, d, g, Agonist-induced Halo-Tag-β-arrestin2 recruitment to b2AR-, M2R- or M5R-NLuc in ΔQ-GRK cells in absence of the ubiquitously expressed GRKs (empty vector (EV)-transfected) and in presence of wild type GRK3 or GRK3-CAAX. The concentration-response curves of the data in **Fig. 3** are shown to allow a direct comparison of the GRK3 and GRK3-CAAX condition. Data are shown as Δ net BRET change (%) of at least n=3 independent experiments ± SEM, normalized to the maximum response with GRK3. **b, e, h,** Normalized BRET data of the highest stimulation of **a, d, g** are displayed as bar graphs and statistical differences were tested using one-way ANOVA, followed by a Tukey's test. **c, f, i,** The Halo-corrected mean Δ net BRET fold changes + SEM of the same dataset (not normalized to the highest stimulated value of the GRK3 WT condition) are presented as bar graphs before (basal) and after stimulation with 10 µM Iso or 100 µM ACh (stimulated). The data were normalized to the basal BRET ratio derived from the EV-transfected condition (dotted line). Statistical differences within one condition between basal and stimulated or between the differently transfected conditions were tested using two-way ANOVA, followed by a Sidak's or Tukey's test respectively. All detailed statistical results are provided in **Suppl. Tab. 12**.

Supplementary Figure 8

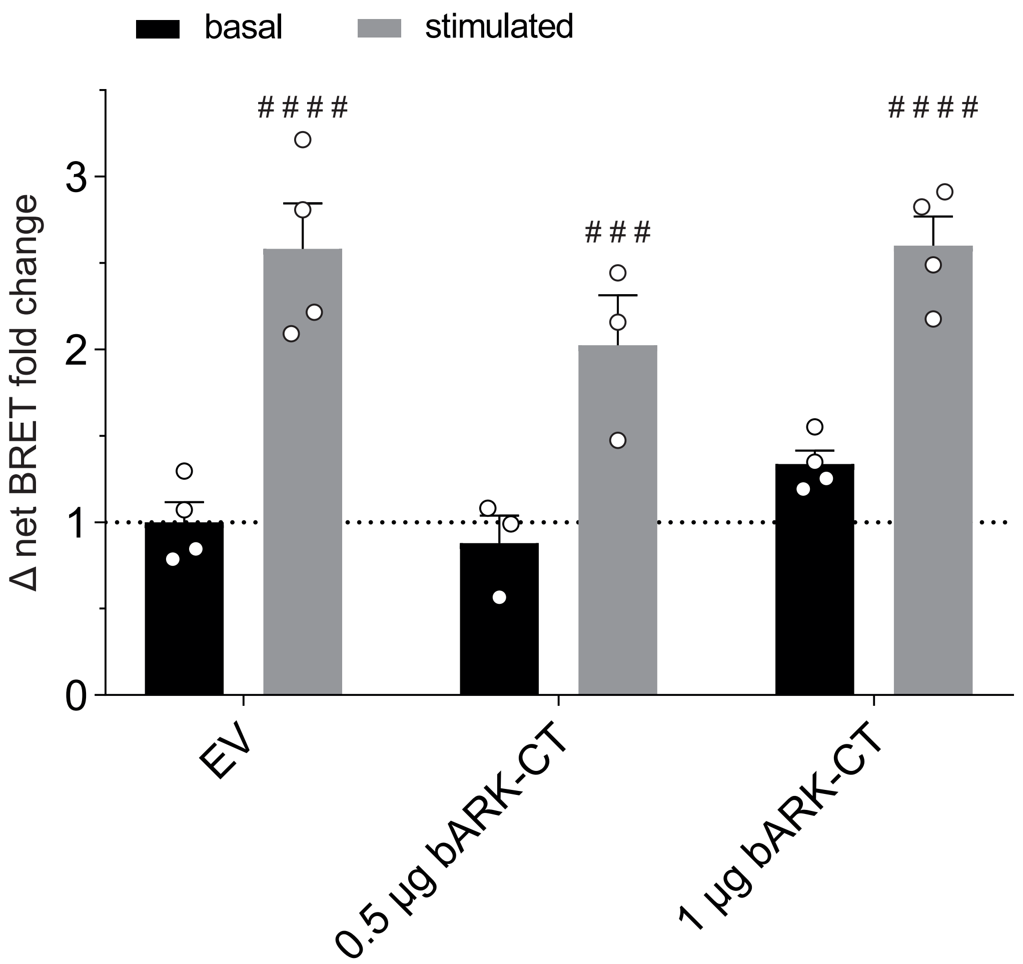

Suppl. Fig. 8: β-arrestin2 recruitment to the muscarinic M5 acetylcholine receptor (M5R) in the presence of bARK-CT. The Halo-corrected mean Δ net BRET fold changes + SEM of the same dataset as shown in **Fig. 4b** (not normalized to the highest stimulated value of the empty vector (EV) condition) are presented as bar graphs before (basal) and after stimulation with 100 µM ACh (stimulated). The data were normalized to the basal BRET ratio derived from the EV-transfected condition (dotted line). Statistical differences within one condition between basal and stimulated (#) or between the differently transfected conditions were tested using two-way ANOVA, followed by a Sidak's or Tukey's test respectively (### $p < 0.001$; #### $p < 0.0001$). All detailed statistical results are provided in **Suppl. Tab. 13**.

Supplementary Figure 9

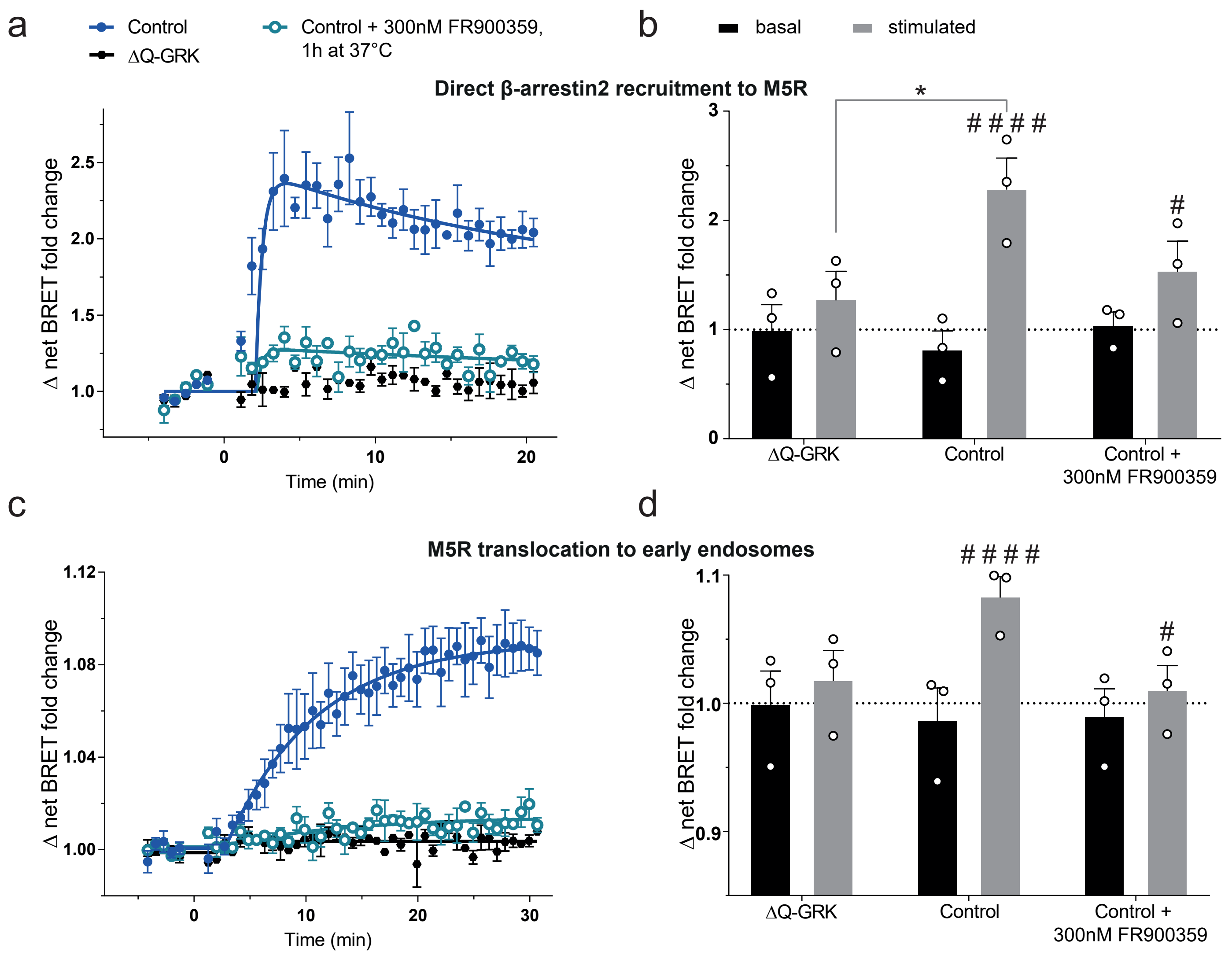

Suppl. Fig. 9: β-arrestin2 recruitment to the muscarinic M5 acetylcholine receptor (M5R) and M5R translocation to early endosomes in presence of FR900359. a, Halo-Tag-β-arrestin2 recruitment to M5R-NLuc was measured in CRISPR/Cas9 HEK293 control cells (Control), expressing all GRKs at endogenous levels, in absence or presence of 300 nM FR900359 or in quadruple GRK2/3/5/6 knockout cells (ΔQ-GRK). Dataset of **Fig. 5b** is shown as Δ net BRET fold changes over time of *n*=3 independent experiments ± SEM. Ligand (100 µM ACh) was added at time point 0 min. **b,** The Halo-corrected mean Δ net BRET fold changes + SEM of the same dataset as shown in **Fig. 5b** (not normalized to the highest stimulated value of Control condition) are presented as bar graphs before (basal) and after stimulation with 100 µM ACh (stimulated). The data were normalized to the basal BRET ratio derived from the ΔQ-GRK condition (dotted line). Statistical differences within one condition between basal and stimulated (#) or between the differently transfected conditions (*) were tested using two-way ANOVA, followed by a Sidak's or Tukey's test respectively (#/* $p < 0.05$; ####/**** $p < 0.0001$). **c,** M5R-NLuc translocation to early endosomes (FYVE-Neongreen) as a measure of internalization was assessed in Control cells in absence or presence of 300 nM FR900359 or in ΔQ-GRK cells. Dataset of **Fig. 5d** is shown as Δ net BRET fold changes over time of *n=3* independent experiments ± SEM. Ligand (100 µM ACh) was added at time point 0 min. **d,** The mean Δ net BRET fold changes + SEM of the same dataset as shown in **Fig. 5d** (not normalized to the highest stimulated value of Control condition) are presented as bar graphs before (basal) and after stimulation with 100 µM ACh (stimulated). Normalization and statistical analysis was performed as described above. All detailed statistical results are provided in **Suppl. Tab. 14**.

Supplementary Table 1: Detailed statistical results of the analysis of the fold change in intensity (over background) in the fluorescence intensity measurements of NeonGreen-coupled GRK2 constructs are shown as presented in **Supplementary Figure 2b**. The fold change in intensity of each condition were compared using one-way ANOVA, followed by a Tukey's test (*ns* not significant; * $p < 0.05$; ** $p < 0.01$; *** $p < 0.001$; **** $p < 0.0001$). For each condition the mean difference, 95% confidence interval (CI) of the difference, and the adjusted *p* value are shown.

conditions	mean diff.	95% CI of diff.	adjusted *p* value
GRK2 vs. GRK2-D110A	-27.93	-119.6 to 63.74	0.9576 ns
GRK2 vs. GRK2-R587Q	-31.2	-122.9 to 60.48	0.9271 ns
GRK2 vs. GRK2-DM	-28.75	-120.4 to 62.92	0.9509 ns
GRK2 vs. GRK2-CAAX	39.43	-52.24 to 131.1	0.8024 ns
GRK2 vs. GRK2-D110A-CAAX	13.48	-78.19 to 105.2	0.9994 ns
GRK2 vs. GRK2-R587Q-CAAX	24.2	-67.47 to 115.9	0.9801 ns
GRK2 vs. GRK2-DM-CAAX	7.433	-84.24 to 99.11	>0.9999 ns
GRK2-D110A vs. GRK2-R587Q	-3.268	-94.94 to 88.40	>0.9999 ns
GRK2-D110A vs. GRK2-DM	-0.819	-92.49 to 90.85	>0.9999 ns
GRK2-D110A vs. GRK2-CAAX	67.36	-24.31 to 159.0	0.2454 ns
GRK2-D110A vs. GRK2-D110A-CAAX	41.41	-50.26 to 133.1	0.7637 ns
GRK2-D110A vs. GRK2-R587Q-CAAX	52.13	-39.54 to 143.8	0.5289 ns
GRK2-D110A vs. GRK2-DM-CAAX	35.36	-56.31 to 127.0	0.8722 ns
GRK2-R587Q vs. GRK2-DM	2.449	-89.22 to 94.12	>0.9999 ns
GRK2-R587Q vs. GRK2-CAAX	70.63	-21.05 to 162.3	0.2022 ns
GRK2-R587Q vs. GRK2-D110A-CAAX	44.68	-47.00 to 136.3	0.6949 ns
GRK2-R587Q vs. GRK2-R587Q-CAAX	55.4	-36.28 to 147.1	0.4584 ns
GRK2-R587Q vs. GRK2-DM-CAAX	38.63	-53.04 to 130.3	0.8173 ns
GRK2-DM vs. GRK2-CAAX	68.18	-23.49 to 159.8	0.234 ns
GRK2-DM vs. GRK2-D110A-CAAX	42.23	-49.44 to 133.9	0.747 ns
GRK2-DM vs. GRK2-R587Q-CAAX	52.95	-38.72 to 144.6	0.5109 ns
GRK2-DM vs. GRK2-DM-CAAX	36.18	-55.49 to 127.9	0.8594 ns
GRK2-CAAX vs. GRK2-D110A-CAAX	-25.95	-117.6 to 65.72	0.9711 ns
GRK2-CAAX vs. GRK2-R587Q-CAAX	-15.23	-106.9 to 76.44	0.9988 ns
GRK2-CAAX vs. GRK2-DM-CAAX	-32	-123.7 to 59.68	0.9179 ns
GRK2-D110A-CAAX vs. GRK2-R587Q-CAAX	10.72	-80.95 to 102.4	0.9999 ns
GRK2-D110A-CAAX vs. GRK2-DM-CAAX	-6.047	-97.72 to 85.63	>0.9999 ns
GRK2-R587Q-CAAX vs. GRK2-DM-CAAX	-16.77	-108.4 to 74.91	0.9977 ns

Supplementary Table 2: Detailed statistical results of the analysis of the Δ net BRET change in β-arrestin2 recruitment to b2AR, mediated by the indicated GRK construct in ΔQ-GRK cells, are shown as presented in **Figure 1h, i**. The Δ net BRET changes at 10 µM Isoprenaline (Iso) of each condition were compared using one-way ANOVA, followed by a Tukey's test (*ns* not significant; * $p < 0.05$; ** $p < 0.01$; *** $p < 0.001$; **** $p < 0.0001$). For each condition the mean difference, 95% confidence interval (CI) of the difference, and the adjusted *p* value are shown.

conditions	mean diff.	95% CI of diff.	adjusted *p* value	
EV vs. GRK2	-78.11	-106 to -50.18	<0.0001	****
EV vs. GRK2-D110A	-82.49	-110.4 to -54.57	<0.0001	****
EV vs. GRK2-R587Q	-9.005	-34.86 to 16.85	0.8052	ns
EV vs. GRK2-D110A,R587Q	-9.693	-35.55 to 16.16	0.762	ns
GRK2 vs. GRK2-D110A	-4.386	-34.24 to 25.47	0.9895	ns
GRK2 vs. GRK2-R587Q	69.1	41.18 to 97.03	<0.0001	****
GRK2 vs. GRK2-D110A,R587Q	68.41	40.49 to 96.34	<0.0001	****
GRK2-D110A vs. GRK2-R587Q	73.49	45.56 to 101.4	<0.0001	****
GRK2-D110A vs. GRK2-D110A,R587Q	72.8	44.87 to 100.7	<0.0001	****
GRK2-R587Q vs. GRK2-D110A,R587Q	-0.6872	-26.54 to 25.17	>0.9999	ns
EV vs. GRK2-CAAX	-78.15	-108.8 to -47.54	<0.0001	****
EV vs. GRK2-D110A-CAAX	-75.07	-105.7 to -44.46	<0.0001	****
EV vs. GRK2-R587Q-CAAX	-60.52	-91.13 to -29.92	0.0002	***
EV vs. GRK2-D110A,R587Q-CAAX	-62.7	-93.31 to -32.1	0.0001	***
GRK2-CAAX vs. GRK2-D110A-CAAX	3.08	-27.53 to 33.69	0.9977	ns
GRK2-CAAX vs. GRK2-R587Q-CAAX	17.62	-12.98 to 48.23	0.4204	ns
GRK2-CAAX vs. GRK2-D110A,R587Q-CAAX	15.45	-15.16 to 46.05	0.5434	ns
GRK2-D110A-CAAX vs. GRK2-R587Q-CAAX	14.54	-16.06 to 45.15	0.597	ns
GRK2-D110A-CAAX vs. GRK2-D110A,R587Q-CAAX	12.37	-18.24 to 42.97	0.7249	ns
GRK2-R587Q-CAAX vs. GRK2-D110A,R587Q-CAAX	-2.177	-32.78 to 28.43	0.9994	ns

Supplementary Table 3: Detailed statistical results of the analysis of the Δ net BRET change in β-arrestin2 recruitment to b2AR, mediated by the indicated GRK2 construct in ΔQ-GRK cells, are shown as presented in **Supplementary Figure 3b, c**. The Δ net BRET changes at 10 µM Isoprenaline (Iso) of each condition were compared using one-way ANOVA, followed by a Tukey's test (*ns* not significant; * $p < 0.05$; ** $p < 0.01$; *** $p < 0.001$; **** $p < 0.0001$). The non-normalized, Halo-corrected mean Δ net BRET fold changes before (basal) and after stimulation with 10 µM Iso (stimulated) were compared between the conditions or within one condition, as indicated. Statistical analysis was performed by two-way ANOVA, followed by a Tukey's or Sidak's test respectively (*ns* not significant; * $p < 0.05$; ** $p < 0.01$; *** $p < 0.001$; **** $p < 0.0001$). For each condition the mean difference, 95% confidence interval (CI) of the difference, and the adjusted *p* value are shown.

Statistical details of Suppl. Fig. 3b

conditions	mean diff.	95% CI of diff.	adjusted *p* value	
EV vs. GRK2	-78.11	-104.4 to -51.81	<0.0001	****
EV vs. GRK2 CAAX	-78.3	-102.6 to -53.95	<0.0001	****
GRK2 vs. GRK2 CAAX	-0.1926	-26.49 to 26.11	0.9998	ns

Statistical details of Suppl. Fig. 3c

comparison of basal and stimulated between conditions

basal	mean diff.	95% CI of diff.	adjusted *p* value	
EV vs. GRK2	-0.07946	-0.7695 to 0.6106	0.9527	ns
EV vs. GRK2 CAAX	-0.1161	-0.755 to 0.5227	0.8866	ns
GRK2 vs. GRK2 CAAX	-0.03669	-0.7268 to 0.6534	0.9897	ns

stimulated	mean diff.	95% CI of diff.	adjusted *p* value	
EV vs. GRK2	-1.058	-1.749 to -0.3684	0.0031	**
EV vs. GRK2 CAAX	-1.002	-1.641 to -0.3627	0.0026	**
GRK2 vs. GRK2 CAAX	0.05685	-0.6332 to 0.7469	0.9754	ns

comparison of basal vs. stimulated within one condition

conditions	mean diff.	95% CI of diff.	adjusted *p* value	
EV	-0.2095	-0.5687 to 0.1498	0.3136	ns
GRK2	-1.188	-1.603 to -0.7737	<0.0001	****
GRK2 CAAX	-1.095	-1.454 to -0.7357	<0.0001	****

Supplementary Table 4: Detailed statistical results of the analysis of the Δ net BRET change in β-arrestin2 recruitment to M2R, mediated by the indicated GRK construct in ΔQ-GRK cells, are shown as presented in **Figure 2a, b**. The Δ net BRET changes at 100 µM Acetylcholine (ACh) of each condition were compa-red using one-way ANOVA, followed by a Tukey‘s test (*ns* not significant; * $p < 0.05$; ** $p < 0.01$; *** $p < 0.001$). For each condition the mean difference, 95% confidence interval (CI) of the difference, and the adjusted *p* value are shown.

conditions	mean diff.	95% CI of diff.	adjusted *p* value	
EV vs. GRK2	-82.71	-116 to -49.41	<0.0001	****
EV vs. GRK2-D110A	-103.9	-137.2 to -70.63	<0.0001	****
EV vs. GRK2-R587Q	-6.587	-39.89 to 26.72	0.9626	ns
EV vs. GRK2-D110A,R587Q	-31.28	-64.59 to 2.019	0.0681	ns
GRK2 vs. GRK2-D110A	-21.22	-54.52 to 12.09	0.2924	ns
GRK2 vs. GRK2-R587Q	76.13	42.82 to 109.4	0.0002	***
GRK2 vs. GRK2-D110A,R587Q	51.43	18.13 to 84.73	0.0034	**
GRK2-D110A vs. GRK2-R587Q	97.34	64.04 to 130.6	<0.0001	****
GRK2-D110A vs. GRK2-D110A,R587Q	72.65	39.34 to 105.9	0.0002	***
GRK2-R587Q vs. GRK2-D110A,R587Q	-24.7	-58 to 8.606	0.1814	ns
EV vs. GRK2-CAAX	-64.07	-116.7 to -11.43	0.0166	*
EV vs. GRK2-D110A-CAAX	-63.34	-116 to -10.7	0.0178	*
EV vs. GRK2-R587Q-CAAX	-59.71	-112.4 to -7.066	0.0252	*
EV vs. GRK2-D110A,R587Q-CAAX	-113.8	-166.4 to -61.16	0.0002	***
GRK2-CAAX vs. GRK2-D110A-CAAX	0.7327	-51.91 to 53.37	>0.9999	ns
GRK2-CAAX vs. GRK2-R587Q-CAAX	4.362	-48.28 to 57	0.9986	ns
GRK2-CAAX vs. GRK2-D110A,R587Q-CAAX	-49.73	-102.4 to 2.915	0.0663	ns
GRK2-D110A-CAAX vs. GRK2-R587Q-CAAX	3.629	-49.01 to 56.27	0.9993	ns
GRK2-D110A-CAAX vs. GRK2-D110A,R587Q-CAAX	-50.46	-103.1 to 2.183	0.0618	ns
GRK2-R587Q-CAAX vs. GRK2-D110A,R587Q-CAAX	-54.09	-106.7 to -1.447	0.0434	*

Supplementary Table 5: Detailed statistical results of the analysis of the Δ net BRET change in β-arrestin2 recruitment to M2R, mediated by the indicated GRK2 construct in ΔQ-GRK cells, are shown as presented in **Supplementary Figure 4b, c**. The Δ net BRET changes at 100 µM ACh of each condition were compared using one-way ANOVA, followed by a Tukey's test (*ns* not significant; * $p < 0.05$; ** $p < 0.01$; *** $p < 0.001$; **** $p < 0.0001$). The non-normalized, Halo-corrected mean Δ net BRET fold changes before (basal) and after stimulation with 100 µM ACh (stimulated) were compared between the conditions or within one condition, as indicated. Statistical analysis was performed by two-way ANOVA, followed by a Tukey's or Sidak's test respectively (*ns* not significant; * $p < 0.05$; ** $p < 0.01$; *** $p < 0.001$; **** $p < 0.0001$). For each condition the mean difference, 95% confidence interval (CI) of the difference, and the adjusted *p* value are shown.

Statistical details of Suppl. Fig. 4b

conditions	mean diff.	95% CI of diff.	adjusted *p* value	
EV vs. GRK2	-82.71	-108.2 to -57.24	0.0001	***
EV vs. GRK2 CAAX	-30.83	-56.3 to -5.353	0.0232	*
GRK2 vs. GRK2 CAAX	51.89	26.42 to 77.36	0.0019	**

Statistical details of Suppl. Fig. 4c

comparison of basal and stimulated between conditions

basal	mean diff.	95% CI of diff.	adjusted *p* value	
EV vs. GRK2	-0.1183	-0.7474 to 0.5108	0.8719	ns
EV vs. GRK2 CAAX	-0.9371	-1.566 to -0.308	0.0049	**
GRK2 vs. GRK2 CAAX	-0.8188	-1.448 to -0.1897	0.0119	*

stimulated	mean diff.	95% CI of diff.	adjusted *p* value	
EV vs. GRK2	-0.8504	-1.48 to -0.2213	0.0093	**
EV vs. GRK2 CAAX	-1.223	-1.852 to -0.5937	0.0006	***
GRK2 vs. GRK2 CAAX	-0.3724	-1.001 to 0.2567	0.2915	ns

comparison of basal vs. stimulated within one condition

conditions	mean diff.	95% CI of diff.	adjusted *p* value	
EV	-0.2422	-0.551 to 0.06652	0.1219	ns
GRK2	-0.9743	-1.283 to -0.6656	0.0001	***
GRK2 CAAX	-0.528	-0.8367 to -0.2192	0.0041	**

Supplementary Table 6: Detailed statistical results of the analysis of the Δ net BRET change in β-arrestin2 recruitment to M5R, mediated by the indicated GRK2 construct in ΔQ-GRK cells, are shown as presented in **Figure 2c, d**. The Δ net BRET changes at 100 μM ACh of each condition were compared using one-way ANOVA, followed by a Tukey's test (*ns* not significant; * $p < 0.05$; ** $p < 0.01$; *** $p < 0.001$; **** $p < 0.0001$). For each condition the mean difference, 95% confidence interval (CI) of the difference, and the adjusted *p* value are shown.

conditions	mean diff.	95% CI of diff.	adjusted *p* value	
EV vs. GRK2	-98.85	-128.9 to -68.81	<0.0001	****
EV vs. GRK2-D110A	-87.82	-119.1 to -56.56	<0.0001	****
EV vs. GRK2-R587Q	-41.19	-72.45 to -9.923	0.0055	**
EV vs. GRK2-D110A,R587Q	-19.88	-51.14 to 11.39	0.3637	ns
GRK2 vs. GRK2-D110A	11.03	-20.24 to 42.29	0.8392	ns
GRK2 vs. GRK2-R587Q	57.66	26.4 to 88.93	<0.0001	****
GRK2 vs. GRK2-D110A,R587Q	78.97	47.71 to 110.2	<0.0001	****
GRK2-D110A vs. GRK2-R587Q	46.64	14.19 to 79.08	0.0022	**
GRK2-D110A vs. GRK2-D110A,R587Q	67.95	35.5 to 100.4	<0.0001	****
GRK2-R587Q vs. GRK2-D110A,R587Q	21.31	-11.13 to 53.75	0.3322	ns
EV vs. GRK2-CAAX	-95.98	-165.3 to -26.7	0.0046	**
EV vs. GRK2-D110A-CAAX	-112.7	-189 to -36.41	0.0026	**
EV vs. GRK2-R587Q-CAAX	-107.4	-176.6 to -38.07	0.0016	**
EV vs. GRK2-D110A,R587Q-CAAX	-113.3	-182.6 to -44.05	0.001	***
GRK2-CAAX vs. GRK2-D110A-CAAX	-16.71	-101.1 to 67.72	0.9728	ns
GRK2-CAAX vs. GRK2-R587Q-CAAX	-11.37	-89.53 to 66.79	0.9913	ns
GRK2-CAAX vs. GRK2-D110A,R587Q-CAAX	-17.36	-95.52 to 60.81	0.9591	ns
GRK2-D110A-CAAX vs. GRK2-R587Q-CAAX	5.336	-79.09 to 89.76	0.9997	ns
GRK2-D110A-CAAX vs. GRK2-D110A,R587Q-CAAX	-0.6486	-85.07 to 83.77	>0.9999	ns
GRK2-R587Q-CAAX vs. GRK2-D110A,R587Q-CAAX	-5.985	-84.15 to 72.18	0.9993	ns

Supplementary Table 7: Detailed statistical results of the analysis of the Δ net BRET change in β-arrestin2 recruitment to M5R, mediated by the indicated GRK2 construct in ΔQ-GRK cells, are shown as presented in **Supplementary Figure 5b, c**. The Δ net BRET changes at 100 µM ACh of each condition were compared using one-way ANOVA, followed by a Tukey's test (*ns* not significant; * $p < 0.05$; ** $p < 0.01$; *** $p < 0.001$; **** $p < 0.0001$). The non-normalized, Halo-corrected mean Δ net BRET fold changes before (basal) and after stimulation with 100 µM ACh (stimulated) were compared between the conditions or within one condition, as indicated. Statistical analysis was performed by two-way ANOVA, followed by a Tukey's or Sidak's test respectively (*ns* not significant; * $p < 0.05$; ** $p < 0.01$; *** $p < 0.001$; **** $p < 0.0001$). For each condition the mean difference, 95% confidence interval (CI) of the difference, and the adjusted *p* value are shown.

Statistical details of Suppl. Fig. 5b

conditions	mean diff.	95% CI of diff.	adjusted *p* value	
EV vs. GRK2	-98.85	-124.3 to -73.39	<0.0001	****
EV vs. GRK2 CAAX	-27.49	-57.34 to 2.361	0.0733	ns
GRK2 vs. GRK2 CAAX	71.36	41.51 to 101.2	<0.0001	****

Statistical details of Suppl. Fig. 5c

comparison of basal and stimulated between conditions

basal	mean diff.	95% CI of diff.	adjusted *p* value	
EV vs. GRK2	0.02301	-0.4865 to 0.5325	0.9932	ns
EV vs. GRK2 CAAX	-0.8273	-1.425 to -0.2298	0.0051	**
GRK2 vs. GRK2 CAAX	-0.8503	-1.448 to -0.2528	0.004	**

stimulated	mean diff.	95% CI of diff.	adjusted *p* value	
EV vs. GRK2	-1.152	-1.662 to -0.6429	<0.0001	****
EV vs. GRK2 CAAX	-1.481	-2.079 to -0.8839	<0.0001	****
GRK2 vs. GRK2 CAAX	-0.329	-0.9264 to 0.2685	0.3755	ns

comparison of basal vs. stimulated within one condition

conditions	mean diff.	95% CI of diff.	adjusted *p* value	
EV	-0.1559	-0.4621 to 0.1503	0.472	ns
GRK2	-1.331	-1.638 to -1.025	<0.0001	****
GRK2 CAAX	-0.81	-1.215 to -0.4049	0.0002	***

Supplementary Table 8: Detailed statistical results of the analysis of the fold change in intensity (over background) in the fluorescence intensity measurements of NeonGreen-coupled GRK3 constructs are shown as presented in **Supplementary Figure 6b**. The fold change in intensity of each condition were compared using one-way ANOVA, followed by a Tukey‘s test (*ns* not significant; * $p < 0.05$; ** $p < 0.01$; *** $p < 0.001$; **** $p < 0.0001$). For each condition the mean difference, 95% confidence interval (CI) of the difference, and the adjusted *p* value are shown.

**conditions**	**mean diff.**	**95% CI of diff.**	**adjusted *p* value**	
GRK3 vs. GRK3-D110A	-1.871	-61.59 to 57.84	>0.9999	ns
GRK3 vs. GRK3-R587Q	-10.34	-70.05 to 49.38	0.9984	ns
GRK3 vs. GRK3-DM	-31.79	-91.50 to 27.93	0.6032	ns
GRK3 vs. GRK3-CAAX	-9.774	-69.49 to 49.94	0.9989	ns
GRK3 vs. GRK3-D110A-CAAX	-2.213	-61.93 to 57.50	>0.9999	ns
GRK3 vs. GRK3-R587Q-CAAX	-4.191	-63.91 to 55.52	>0.9999	ns
GRK3 vs. GRK3-DM-CAAX	-39.89	-99.60 to 19.83	0.3441	ns
GRK3-D110A vs. GRK3-R587Q	-8.464	-68.18 to 51.25	0.9996	ns
GRK3-D110A vs. GRK3-DM	-29.92	-89.63 to 29.80	0.6674	ns
GRK3-D110A vs. GRK3-CAAX	-7.903	-67.62 to 51.81	0.9997	ns
GRK3-D110A vs. GRK3-D110A-CAAX	-0.3417	-60.06 to 59.37	>0.9999	ns
GRK3-D110A vs. GRK3-R587Q-CAAX	-2.32	-62.03 to 57.40	>0.9999	ns
GRK3-D110A vs. GRK3-DM-CAAX	-38.02	-97.73 to 21.70	0.3982	ns
GRK3-R587Q vs. GRK3-DM	-21.45	-81.17 to 38.26	0.9063	ns
GRK3-R587Q vs. GRK3-CAAX	0.5616	-59.15 to 60.28	>0.9999	ns
GRK3-R587Q vs. GRK3-D110A-CAAX	8.122	-51.59 to 67.84	0.9997	ns
GRK3-R587Q vs. GRK3-R587Q-CAAX	6.144	-53.57 to 65.86	>0.9999	ns
GRK3-R587Q vs. GRK3-DM-CAAX	-29.55	-89.27 to 30.16	0.6798	ns
GRK3-DM vs. GRK3-CAAX	22.02	-37.70 to 81.73	0.8949	ns
GRK3-DM vs. GRK3-D110A-CAAX	29.58	-30.14 to 89.29	0.6789	ns
GRK3-DM vs. GRK3-R587Q-CAAX	27.6	-32.12 to 87.31	0.7441	ns
GRK3-DM vs. GRK3-DM-CAAX	-8.097	-67.81 to 51.62	0.9997	ns
GRK3-CAAX vs. GRK3-D110A-CAAX	7.561	-52.15 to 67.28	0.9998	ns
GRK3-CAAX vs. GRK3-R587Q-CAAX	5.583	-54.13 to 65.30	>0.9999	ns
GRK3-CAAX vs. GRK3-DM-CAAX	-30.11	-89.83 to 29.60	0.6607	ns
GRK3-D110A-CAAX vs. GRK3-R587Q-CAAX	-1.978	-61.69 to 57.74	>0.9999	ns
GRK3-D110A-CAAX vs. GRK3-DM-CAAX	-37.67	-97.39 to 22.04	0.4085	ns
GRK3-R587Q-CAAX vs. GRK3-DM-CAAX	-35.7	-95.41 to 24.02	0.471	ns

Supplementary Table 9: Detailed statistical results of the analysis of the Δ net BRET change in β-arrestin2 recruitment to b2AR, mediated by the indicated GRK3 construct in ΔQ-GRK cells, are shown as presented in **Figure 3a, b**. The Δ net BRET changes at 10 µM Iso of each condition were compared using one-way ANOVA, followed by a Tukey's test (*ns* not significant; * p < 0.05; ** p < 0.01; *** p < 0.001; **** p < 0.0001). For each condition the mean difference, 95% confidence interval (CI) of the difference, and the adjusted p value are shown.

**conditions**	**mean diff.**	**95% CI of diff.**	**adjusted *p* value**	
EV vs. GRK3	-70.23	-115.2 to -25.21	0.002	**
EV vs. GRK3-D110A	-64.53	-113.1 to -15.9	0.0075	**
EV vs. GRK3-R587Q	0.298	-44.72 to 45.31	>0.9999	ns
EV vs. GRK3-D110A,R587Q	4.095	-40.92 to 49.11	0.9984	ns
GRK3 vs. GRK3-D110A	5.7	-42.92 to 54.32	0.9957	ns
GRK3 vs. GRK3-R587Q	70.52	25.51 to 115.5	0.0019	**
GRK3 vs. GRK3-D110A,R587Q	74.32	29.31 to 119.3	0.0012	**
GRK3-D110A vs. GRK3-R587Q	64.82	16.2 to 113.4	0.0073	**
GRK3-D110A vs. GRK3-D110A,R587Q	68.62	20 to 117.2	0.0046	**
GRK3-R587Q vs. GRK3-D110A,R587Q	3.797	-41.22 to 48.81	0.9988	ns
EV vs. GRK3-CAAX	-65.39	-91.19 to -39.58	<0.0001	****
EV vs. GRK3-D110A-CAAX	-50.44	-76.24 to -24.63	0.0002	***
EV vs. GRK3-R587Q-CAAX	-40.91	-68.78 to -13.03	0.0033	**
EV vs. GRK3-D110A,R587Q-CAAX	-17.57	-43.37 to 8.24	0.2649	ns
GRK3-CAAX vs. GRK3-D110A-CAAX	14.95	-10.86 to 40.75	0.4085	ns
GRK3-CAAX vs. GRK3-R587Q-CAAX	24.48	-3.393 to 52.35	0.098	ns
GRK3-CAAX vs. GRK3-D110A,R587Q-CAAX	47.82	22.02 to 73.63	0.0004	***
GRK3-D110A-CAAX vs. GRK3-R587Q-CAAX	9.531	-18.34 to 37.4	0.8207	ns
GRK3-D110A-CAAX vs. GRK3-D110A,R587Q-CAAX	32.87	7.067 to 58.68	0.0103	*
GRK3-R587Q-CAAX vs. GRK3-D110A,R587Q-CAAX	23.34	-4.531 to 51.21	0.1219	ns

Supplementary Table 10: Detailed statistical results of the analysis of the Δ net BRET change in β-arrestin2 recruitment to M2R, mediated by the indicated GRK3 construct in ΔQ-GRK cells, are shown as presented in **Figure 3c, d**. The Δ net BRET changes at 100 µM ACh of each condition were compared using one-way ANOVA, followed by a Tukey's test (*ns* not significant; * $p < 0.05$; ** $p < 0.01$; *** $p < 0.001$; **** $p < 0.0001$). For each condition the mean difference, 95% confidence interval (CI) of the difference, and the adjusted *p* value are shown.

conditions	mean diff.	95% CI of diff.	adjusted *p* value	
EV vs. GRK3	-82.13	-133.2 to -31.1	0.0015	**
EV vs. GRK3-D110A	-62.4	-113.4 to -11.37	0.0138	*
EV vs. GRK3-R587Q	11.8	-39.23 to 62.84	0.9482	ns
EV vs. GRK3-D110A,R587Q	-13.03	-68.15 to 42.09	0.9441	ns
GRK3 vs. GRK3-D110A	19.74	-31.29 to 70.77	0.7487	ns
GRK3 vs. GRK3-R587Q	93.94	42.91 to 145	0.0004	***
GRK3 vs. GRK3-D110A,R587Q	69.1	13.98 to 124.2	0.0116	*
GRK3-D110A vs. GRK3-R587Q	74.2	23.17 to 125.2	0.0036	**
GRK3-D110A vs. GRK3-D110A,R587Q	49.37	-5.754 to 104.5	0.0893	ns
GRK3-R587Q vs. GRK3-D110A,R587Q	-24.84	-79.95 to 30.28	0.6352	ns
EV vs. GRK3-CAAX	-77.48	-123.4 to -31.53	0.0011	**
EV vs. GRK3-D110A-CAAX	-61.54	-107.5 to -15.59	0.0074	**
EV vs. GRK3-R587Q-CAAX	-48.05	-97.68 to 1.586	0.0596	ns
EV vs. GRK3-D110A,R587Q-CAAX	-21.72	-71.35 to 27.92	0.6512	ns
GRK3-CAAX vs. GRK3-D110A-CAAX	15.94	-30.01 to 61.89	0.8074	ns
GRK3-CAAX vs. GRK3-R587Q-CAAX	29.44	-20.2 to 79.07	0.3799	ns
GRK3-CAAX vs. GRK3-D110A,R587Q-CAAX	55.77	6.133 to 105.4	0.025	*
GRK3-D110A-CAAX vs. GRK3-R587Q-CAAX	13.49	-36.14 to 63.13	0.9078	ns
GRK3-D110A-CAAX vs. GRK3-D110A,R587Q-CAAX	39.82	-9.809 to 89.46	0.144	ns
GRK3-R587Q-CAAX vs. GRK3-D110A,R587Q-CAAX	26.33	-26.73 to 79.39	0.5438	ns

Supplementary Table 11: Detailed statistical results of the analysis of the Δ net BRET change in β-arrestin2 recruitment to M5R, mediated by the indicated GRK3 construct in ΔQ-GRK cells, are shown as presented in **Figure 3e , f** . The Δ net BRET changes at 100 μM ACh of each condition were compared using one-way ANOVA, followed by a Tukey‘s test (*ns* not significant; * $p < 0.05$; ** $p < 0.01$; *** $p < 0.001$; **** $p < 0.0001$). For each condition the mean difference, 95% confidence interval (CI) of the difference, and the adjusted *p* value are shown.

conditions	mean diff.	95% CI of diff.	adjusted *p* value	
EV vs. GRK3	-98.03	-129.3 to -66.79	<0,0001	****
EV vs. GRK3-D110A	-86.07	-119.0 to -53.19	<0,0001	****
EV vs. GRK3-R587Q	-41.94	-74.83 to -9.054	0.008	**
EV vs. GRK3-D110A,R587Q	-8.129	-41.02 to 24.76	0.9471	ns
GRK3 vs. GRK3-D110A	11.96	-22.05 to 45.97	0.8344	ns
GRK3 vs. GRK3-R587Q	56.09	22.08 to 90.10	0.0006	***
GRK3 vs. GRK3-D110A,R587Q	89.91	55.90 to 123.9	<0,0001	****
GRK3-D110A vs. GRK3-R587Q	44.13	8.610 to 79.65	0.01	*
GRK3-D110A vs. GRK3-D110A,R587Q	77.94	42.42 to 113.5	<0,0001	****
GRK3-R587Q vs. GRK3-D110A,R587Q	33.81	-1.710 to 69.33	0.0674	ns
EV vs. GRK3-CAAX	-96.81	-161.8 to -31.86	0.0022	**
EV vs. GRK3-D110A-CAAX	-114.5	-179.4 to -49.53	0.0004	***
EV vs. GRK3-R587Q-CAAX	-120	-184.9 to -55.03	0.0002	***
EV vs. GRK3-D110A,R587Q-CAAX	-72.58	-137.5 to -7.630	0.0243	*
GRK3-CAAX vs. GRK3-D110A-CAAX	-17.67	-90.95 to 55.60	0.9468	ns
GRK3-CAAX vs. GRK3-R587Q-CAAX	-23.17	-96.45 to 50.10	0.8708	ns
GRK3-CAAX vs. GRK3-D110A,R587Q-CAAX	24.23	-49.05 to 97.50	0.852	ns
GRK3-D110A-CAAX vs. GRK3-R587Q-CAAX	-5.502	-78.78 to 67.77	0.9994	ns
GRK3-D110A-CAAX vs. GRK3-D110A,R587Q-CAAX	41.9	-31.38 to 115.2	0.4421	ns
GRK3-R587Q-CAAX vs. GRK3-D110A,R587Q-CAAX	47.4	-25.87 to 120.7	0.3254	ns

Supplementary Table 12: Detailed statistical results of the analysis of the Δ net BRET change in β-arrestin2 recruitment to b2AR, M2R and M5R, mediated by the indicated GRK3 construct in ΔQ-GRK cells, are shown as presented in **Supplementary Figure 7**. The Δ net BRET changes at highest ligand concentration of each condition were compared using one-way ANOVA (**Suppl. Fig. 7b, e, h**), followed by a Tukey's test (*ns* not significant; * $p < 0.05$; ** $p < 0.01$; *** $p < 0.001$; **** $p < 0.0001$). The non-normalized, Halo-corrected mean Δ net BRET fold changes before (basal) and after stimulation with highest ligand concentration (stimulated) were compared between the conditions or within one condition (**Suppl. Fig. 7c, f, i**), as indicated. Statistical analysis was performed by two-way ANOVA, followed by a Tukey's or Sidak's test respectively (*ns* not significant; * $p < 0.05$; ** $p < 0.01$; *** $p < 0.001$; **** $p < 0.0001$). For each condition the mean difference, 95% confidence interval (CI) of the difference, and the adjusted *p* value are shown.

b2AR

Statistical details of Suppl. Fig. 7b

conditions	mean diff.	95% CI of diff.	adjusted *p* value
EV vs. GRK3	-70.23	-109.4 to -31.04	0.0019 **
EV vs. GRK3 CAAX	-56.22	-95.41 to -17.04	0.0078 **
GRK3 vs. GRK3 CAAX	14	-25.18 to 53.19	0.5964 ns

Statistical details of Suppl. Fig. 7c

comparison of basal and stimulated between conditions

basal	mean diff.	95% CI of diff.	adjusted *p* value
EV vs. GRK3	-0.05886	-0.4522 to 0.3345	0.9231 ns
EV vs. GRK3 CAAX	-0.05543	-0.4488 to 0.3379	0.9315 ns
GRK3 vs. GRK3 CAAX	0.003428	-0.3899 to 0.3968	0.9997 ns

stimulated	mean diff.	95% CI of diff.	adjusted *p* value
EV vs. GRK3	-0.8634	-1.257 to -0.47	<0.0001 ****
EV vs. GRK3 CAAX	-0.6237	-1.017 to -0.2303	0.0021 **
GRK3 vs. GRK3 CAAX	0.2397	-0.1537 to 0.6331	0.29 ns

comparison of basal vs. stimulated within one condition

conditions	mean diff.	95% CI of diff.	adjusted *p* value
EV	-0.2476	-0.561 to 0.06585	0.1326 ns
GRK3	-1.052	-1.366 to -0.7387	<0.0001 ****
GRK3 CAAX	-0.8158	-1.129 to -0.5024	<0.0001 ****

M2R

Statistical details of Suppl. Fig. 7e

conditions	mean diff.	95% CI of diff.	adjusted *p* value
EV vs. GRK3	-82.13	-133.1 to -31.2	0.0038 **
EV vs. GRK3 CAAX	-61.49	-112.4 to -10.56	0.0203 *
GRK3 vs. GRK3 CAAX	20.65	-30.29 to 71.58	0.5198 ns

Statistical details of Suppl. Fig. 7f

comparison of basal and stimulated between conditions

basal	mean diff.	95% CI of diff.	adjusted *p* value
EV vs. GRK3	-0.02757	-0.4106 to 0.3555	0.9816 ns
EV vs. GRK3 CAAX	0.1415	-0.2416 to 0.5245	0.6213 ns
GRK3 vs. GRK3 CAAX	0.169	-0.214 to 0.5521	0.5109 ns

stimulated	mean diff.	95% CI of diff.	adjusted *p* value
EV vs. GRK3	-0.9904	-1.373 to -0.6073	<0.0001 ****
EV vs. GRK3 CAAX	-0.5045	-0.8875 to -0.1214	0.0093 **
GRK3 vs. GRK3 CAAX	0.4859	0.1028 to 0.8689	0.0121 *

comparison of basal vs. stimulated within one condition

conditions	mean diff.	95% CI of diff.	adjusted *p* value
EV	-0.2365	-0.455 to -0.01798	0.0341 *
GRK3	-1.199	-1.418 to -0.9808	<0.0001 ****
GRK3 CAAX	-0.8824	-1.101 to -0.6639	<0.0001 ****

M5R

Statistical details of Suppl. Fig. 7h

conditions	mean diff.	95% CI of diff.	adjusted *p* value
EV vs. GRK3	-98.37	-130.2 to -66.55	<0.0001 ****
EV vs. GRK3 CAAX	-50.56	-89.53 to -11.59	0.0121 *
GRK3 vs. GRK3 CAAX	47.81	8.843 to 86.77	0.017 *

Statistical details of Suppl. Fig. 7i

comparison of basal and stimulated between conditions

basal	mean diff.	95% CI of diff.	adjusted *p* value
EV vs. GRK3	-0.01621	-0.4838 to 0.4514	0.9959 ns
EV vs. GRK3 CAAX	-0.322	-0.8946 to 0.2507	0.3547 ns
GRK3 vs. GRK3 CAAX	-0.3058	-0.8784 to 0.2669	0.391 ns

stimulated	mean diff.	95% CI of diff.	adjusted *p* value
EV vs. GRK3	-1.216	-1.684 to -0.7485	<0.0001 ****
EV vs. GRK3 CAAX	-1.117	-1.69 to -0.5444	0.0002 ***
GRK3 vs. GRK3 CAAX	0.099	-0.4737 to 0.6717	0.9028 ns

comparison of basal vs. stimulated within one condition

conditions	mean diff.	95% CI of diff.	adjusted *p* value
EV	-0.1512	-0.3849 to 0.08257	0.2672 ns
GRK3	-1.351	-1.585 to -1.117	<0.0001 ****
GRK3 CAAX	-0.9462	-1.277 to -0.6157	<0.0001 ****

Supplementary Table 13: Detailed statistical results of the analysis of the Δ net BRET change in β-arrestin2 recruitment to M5R, in presence of the indicated amount of bARK-CT in CRISPR/Cas9 Control cells, are shown as presented in **Figure 4b** and **Supplementary Figure 8**. The Δ net BRET changes at 100 µM ACh of each condition were compared using one-way ANOVA (**Fig. 4b**), followed by a Tukey's test (*ns* not significant; * $p < 0.05$; ** $p < 0.01$; *** $p < 0.001$; **** $p < 0.0001$). The non-normalized, Halo-corrected mean Δ net BRET fold changes before (basal) and after stimulation with 100 µM ACh (stimulated) were compared between the conditions or within one condition, as indicated. Statistical analysis was performed by two-way ANOVA (**Suppl. Fig. 8**), followed by a Tukey's or Sidak's test respectively (*ns* not significant; * $p < 0.05$; ** $p < 0.01$; *** $p < 0.001$; **** $p < 0.0001$). For each condition the mean difference, 95% confidence interval (CI) of the difference, and the adjusted *p* value are shown.

Statistical details of Fig. 4b

conditions	mean diff.	95% CI of diff.	adjusted *p* value
EV vs. 0.5 µg bARK-CT	15.9	-8.519 to 40.33	0.2116 ns
EV vs. 1 µg bARK-CT	43.84	21.23 to 66.45	0.0014 **
0.5 µg bARK-CT vs. 1 µg bARK-CT	27.93	3.509 to 52.36	0.0275 *

Statistical details of Suppl. Fig. 8

comparison of basal and stimulated between conditions

basal	mean diff.	95% CI of diff.	adjusted *p* value
EV vs. 0.5 µg bARK-CT	0.1208	-0.5849 to 0.8265	0.8987 ns
EV vs. 1 µg bARK-CT	-0.3362	-0.9896 to 0.3171	0.4008 ns
0.5 µg bARK-CT vs. 1 µg bARK-CT	-0.457	-1.163 to 0.2487	0.2463 ns

stimulated	mean diff.	95% CI of diff.	adjusted *p* value
EV vs. 0.5 µg bARK-CT	0.5567	-0.149 to 1.262	0.1358 ns
EV vs. 1 µg bARK-CT	-0.01848	-0.6718 to 0.6349	0.9971 ns
0.5 µg bARK-CT vs. 1 µg bARK-CT	-0.5752	-1.281 to 0.1305	0.1207 ns

comparison of basal vs. stimulated within one condition

conditions	mean diff.	95% CI of diff.	adjusted *p* value
EV	-1.582	-1.962 to -1.201	<0.0001 ****
0.5 µg bARK-CT	-1.146	-1.585 to -0.7065	0.0002 ***
1 µg bARK-CT	-1.264	-1.644 to -0.8835	<0.0001 ****

Supplementary Table 14: Detailed statistical results of the analysis of the Δ net BRET change in β-arrestin2 recruitment to M5R in CRISPR/Cas9 Control cells, in absence or presence of the indicated amount of FR900359, or in ΔQ-GRK cells is shown as presented in **Figure 5** and **Supplementary Figure 9**. The Δ net BRET changes at 100 µM ACh of each condition were compared using one-way ANOVA (**Fig. 5b, d**), followed by a Tukey's test (*ns* not significant; * $p < 0.05$; ** $p < 0.01$; *** $p < 0.001$; **** $p < 0.0001$). The non-normalized, Halo-corrected mean Δ net BRET fold changes before (basal) and after stimulation with 100 µM ACh (stimulated) were compared between the conditions or within one condition, as indicated. Statistical analysis was performed by two-way ANOVA (**Suppl. Fig. 9b, d**), followed by a Tukey's or Sidak's test respectively (*ns* not significant; * $p < 0.05$; ** $p < 0.01$; *** $p < 0.001$; **** $p < 0.0001$). For each condition the mean difference, 95% confidence interval (CI) of the difference, and the adjusted *p* value are shown.

Direct β-arrestin2 recruitment to M5R

Statistical details of Fig. 5b

conditions	mean diff.	95% CI of diff.	adjusted *p* value	
Control vs. Control + 300nM FR900359	85.37	49.65 to 121.1	0.0008	***
Control vs. ΔQ-GRK	94.98	59.26 to 130.7	0.0004	***
Control + 300nM FR900359 vs. ΔQ-GRK	9.612	-26.11 to 45.33	0.7022	ns

Statistical details of Suppl. Fig. 9b

comparison of basal and stimulated between conditions

basal	mean diff.	95% CI of diff.	adjusted *p* value	
ΔQ-GRK vs. Control	0.1783	-0.6668 to 1.023	0.842	ns
ΔQ-GRK vs. Control + 300nM FR900359	-0.04951	-0.8946 to 0.7955	0.9866	ns
Control vs. Control + 300nM FR900359	-0.2278	-1.073 to 0.6173	0.7571	ns

stimulated	mean diff.	95% CI of diff.	adjusted *p* value	
ΔQ-GRK vs. Control	-1.014	-1.859 to -0.1691	0.0193	*
ΔQ-GRK vs. Control + 300nM FR900359	-0.2634	-1.108 to 0.5817	0.6914	ns
Control vs. Control + 300nM FR900359	0.7507	-0.09431 to 1.596	0.0838	ns

comparison of basal vs. stimulated within one condition

conditions	mean diff.	95% CI of diff.	adjusted *p* value	
DQ-GRK	-0.2813	-0.6589 to 0.0963	0.1441	ns
Control	-1.474	-1.851 to -1.096	<0.0001	****
Control + FR	-0.4952	-0.8728 to -0.1176	0.0153	*

M5R translocation to early endosomes

Statistical details of Fig. 5d

conditions	mean diff.	95% CI of diff.	adjusted *p* value	
Control vs. Control + 300nM FR900359	81.28	45.15 to 117.4	0.0011	**
Control vs. ΔQ-GRK	99.57	63.44 to 135.7	0.0004	***
Control + 300nM FR900359 vs. ΔQ-GRK	18.29	-17.84 to 54.43	0.3339	ns

Statistical details of Suppl. Fig. 9d

comparison of basal and stimulated between conditions

basal	mean diff.	95% CI of diff.	adjusted *p* value	
ΔQ-GRK vs. Control	0.01237	-0.06847 to 0.09321	0.9129	ns
ΔQ-GRK vs. Control + 300nM FR900359	0.009443	-0.07139 to 0.09028	0.9481	ns
Control vs. Control + 300nM FR900359	-0.002927	-0.08376 to 0.07791	0.9949	ns

stimulated	mean diff.	95% CI of diff.	adjusted *p* value	
ΔQ-GRK vs. Control	-0.06501	-0.1458 to 0.01583	0.1221	ns
ΔQ-GRK vs. Control + 300nM FR900359	0.007912	-0.07293 to 0.08875	0.9632	ns
Control vs. Control + 300nM FR900359	0.07292	-0.007919 to 0.1538	0.0787	ns

comparison of basal vs. stimulated within one condition

conditions	mean diff.	95% CI of diff.	adjusted *p* value	
ΔQ-GRK	-0.01852	-0.03774 to 0.0007038	0.058	ns
Control	-0.0959	-0.1151 to -0.07667	<0.0001	****
Control + 300nM FR900359	-0.02005	-0.03927 to -0.0008274	0.0421	*
