# Supplementary material for: GRK specificity and Gβγ dependency determines the potential of a GPCR for arrestin-biased agonism
Source: Commun Biol. 2024 Jul 3;7:802. doi: 10.1038/s42003-024-06490-1 (PMC11220067; doi:10.1038/s42003-024-06490-1)
Supplement: Supplementary file 3 — Description of additional supplementary files [file 42003_2024_6490_MOESM3_ESM.pdf]

# Description of Additional Supplementary Files

**File name:** Supplementary Data 1

**Description:** Source data behind Fig.1

**File name:** Supplementary Data 2

**Description:** Source data behind Fig.2

**File name:** Supplementary Data 3

**Description:** Source data behind Fig.3

**File name:** Supplementary Data 4

**Description:** Source data behind Fig.4

**File name:** Supplementary Data 5

**Description:** Source data behind Fig.5
